# Resolution of Localized Chronic Periodontitis Associated with Longstanding Calculus Deposits

**DOI:** 10.1155/2014/391503

**Published:** 2014-05-05

**Authors:** Pin-Chuang Lai, John D. Walters

**Affiliations:** Division of Periodontology, College of Dentistry, The Ohio State University Wexner Medical Center, 305 West 12th Avenue, Columbus, OH 43210, USA

## Abstract

This report, which is based on nonstandardized serial radiographs obtained over a period of 15 years, documents a case of localized chronic periodontitis associated with progressive deposition of calculus on the distal aspect of a mandibular second molar. The site was treated by scaling and root planing, followed by a course of adjunctive systemic azithromycin. Treatment yielded favorable reductions in probing depth and clinical inflammation, leaving only few isolated sites with pockets no deeper than 4 mm. Two years after completion of active treatment, there was radiographic evidence of increased bone density distal to the second molar.

## 1. Introduction

While it is well established that dental plaque is the primary etiological factor in the pathogenesis of periodontal diseases, epidemiological studies clearly indicate the association between dental calculus and periodontitis [1, 2]. Dental calculus is calcified bacterial plaque. Supragingival and subgingival calculus differ in degrees of mineralization, but they are both typically covered by a layer of bacterial plaque [3, 4]. Although their close relationship with periodontal pathogens and bacterial by-products makes it somewhat difficult to investigate the etiological role of dental calculus alone in periodontitis, it is widely accepted that calculus is a local contributory factor [5]. The rough surface and porous structure of calculus provide an ideal substrate for bacterial colonization and serve as a reservoir for toxic bacterial components and antigen [6]. Moreover, studies have identified viable bacteria within supra- and subgingival calculus, including* Porphyromonas gingivalis*,* Treponema denticola*, and* Aggregatibacter actinomycetemcomitans* [7–9]. Calcifying nanoparticles, the calcified self-propagating entities that are found in dental calculus may contribute to the formation of calculus and pathogenic calcification of epithelial cells [10]. If left untreated, localized periodontal inflammation can persist, leading to breakdown of supporting tissues. Therefore, removal of subgingival plaque and calculus is a necessity for successful periodontal therapy.

While supragingival calculus can be observed through visual examination, clinical detection of subgingival calculus replies on tactile exploration of tooth surfaces with an explorer. Dental calculus on interproximal surfaces may be revealed by radiographs, although the sensitivity of detection differs between radiographic projections [11]. While periapical radiographs are superior to other radiographs in identifying calculus, they only detect 43.8% of the proximal surfaces where deposits were verified visually after extraction [12]. Advanced technologies, including dental endoscopes [13], fiber-optic probes [14], autofluorescence [15], and lasers [16], have been introduced to better detect subgingival calculus. Although it is difficult to completely remove subgingival calculus by scaling and root planing (SRP) [17, 18], periodontal healing appears to occur even in the presence of microscopically visible calculus [19]. Initial periodontal therapy usually results in significant clinical improvement and change of subgingival microbial flora [20, 21]. This report documents the development and treatment of a case of localized chronic periodontitis associated with progressive deposition of calculus on a mandibular second molar over a period of more than 15 years.

## 2. Case Description

A 59-year-old Caucasian female presented in April 2008 for a consultation. She had a history of osteopenia and had been treated with alendronate (Fosamax) for 3 years. Until recently, her history of dental treatment had been uncomplicated. Her third molars had been extracted and four of her permanent teeth had been restored with amalgams. Throughout her life, she had visited her general dentist for semiannual recall appointments. Her dental hygienist and dentist had recently detected periodontal pockets around her maxillary and mandibular right molars. They referred her to a periodontist, who recommended extraction of teeth 17 and 47 (FDI) and possible surgical treatment of the upper left molars. Since she had a history of bisphosphonate use, she was concerned about the potential for development of osteonecrosis of the jaw (ONJ) after the extractions [22]. This motivated her to seek a second opinion to explore options for nonsurgical treatment.

**Figure 1:**
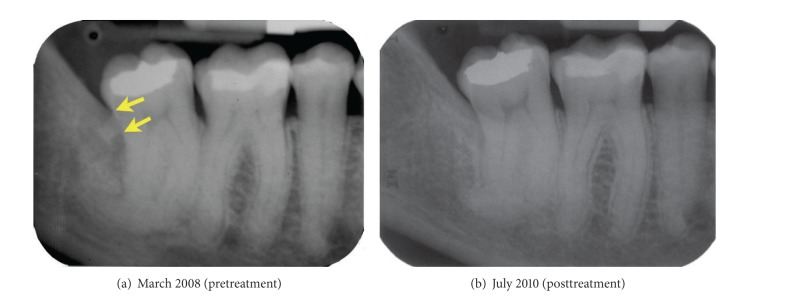
Periapical radiographs of the mandibular right posterior teeth. Radiograph (a) was obtained one month prior to the patient's initial consultation appointment. Arrows indicate the apical and occlusal borders of the calculus deposit. Radiograph (b) was obtained two years after completion of active periodontal treatment.

The patient brought duplicates of periapical radiographs that had been obtained one month earlier by her general dentist. The radiographs revealed the presence of moderate periodontal bone loss around teeth 17 and 16 and early to moderate periodontal bone loss around 27 and on the distal aspect of 37 (not shown). There was severe periodontal bone loss on the distal aspect of 47, which had a pronounced root dilaceration (Figure 1(a)). In addition, there was a radiopaque mass associated with the distal aspect of 47, near the level of the cementoenamel junction. The size and shape of this mass suggested that it was either calculus or a fragment of a third molar root. The patient's oral hygiene was good and clinical signs of inflammation around the premolars and anterior teeth were minimal. However, there were probing depths of 5 to 6 mm on the mesial aspect of 17 and the distal aspect of 16 and probing depths of 7 mm on the direct palatal and distopalatal aspects of 27. Tooth 47 had 5 mm depths on its distofacial and direct lingual aspects and a 10 mm depth on the distolingual. While all of these sites exhibited bleeding on probing, the distal and direct lingual aspects of 47 were the most clinically inflamed. To help determine the identity of the distal radiopaque mass, we requested all available radiographs from dentists who had previously treated the patient.

**Figure 2:**
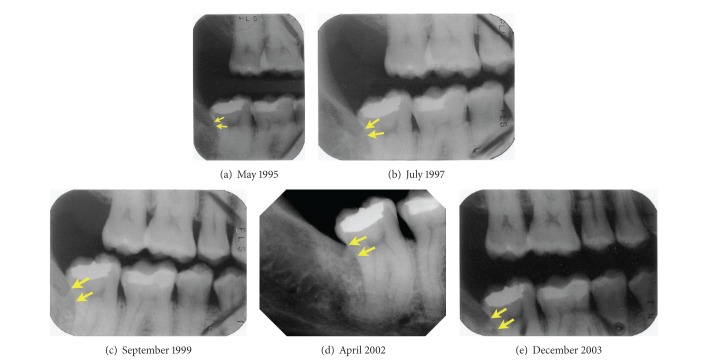
Radiographs of the right molars, taken approximately 13 years (a), 11 years (b), 9 years (c), 6 years (d), and 4.5 years (e) prior to periodontal treatment. The apical and occlusal borders of the calculus are indicated by arrows.

A bitewing film of the right molars taken 13 years earlier provided evidence that the radiopaque mass distal to 47 was calculus (Figure 2(a)). At that time, the calculus deposit was considerably smaller and there were signs of early retromolar bone loss. The calculus deposit enlarged during the next two years, but there was no evidence that bone loss progressed (Figure 2(b)). During the following two-year period, there was further enlargement of the calculus and progression to a moderate degree of bone loss distal to tooth 47 (Figure 2(c)). In a periapical radiograph taken 2.5 years later (6 years prior to the initial consultation), the calculus appeared to have increased in size and distal bone loss had progressed (Figure 2(d)). A bitewing film obtained 1.5 years later suggested that the calculus had not enlarged significantly since the previous radiograph but was not useful for monitoring changes in the degree of bone loss (Figure 2(e)).

Based on the radiographic and clinical findings and the patient's concerns about ONJ, a nonsurgical treatment plan was developed to address the patient's localized severe chronic periodontitis. There was concern about the potential for morbidity related to extraction of 17 and 47, since the roots of 47 were dilacerated. Teeth 17, 16, 27, and 37 were scaled and root planed with curettes and an ultrasonic scaler, followed by adjunctive treatment with a five-day course of systemic azithromycin. Azithromycin was prescribed to help enhance attachment gain and improve the odds of avoiding periosteum-exposing periodontal surgery. The patient was instructed to use an end-tuft brush to remove plaque from her second molars. In the event this initial treatment failed to reduce probing depths and inflammation in an acceptable manner, there was a contingency plan that included localized periodontal surgery.

At the reevaluation appointment five weeks later, the patient presented with a high standard of oral hygiene. There were no sites that bled upon probing or had probing depths greater than 4 mm. The 4 mm probing depths were associated with the distolingual aspect of 27 and the distofacial and distolingual aspect of 47. Given these findings, surgical treatment was not indicated. The patient was scheduled for periodontal maintenance therapy every three months after completion of active treatment. Periodontal probing depths were recorded at the 12-month and 24-month maintenance appointments. The patient's periodontal probing depths and bleeding upon probing were essentially unchanged at these visits. A follow-up radiograph taken at the 24-month recall confirmed that tooth 47 was free of visible calculus deposits and provided evidence of increased density of the bone on the distal aspect of 47 (Figure 1(b)).

## 3. Discussion

This report documents the progressive deposition of subgingival calculus over a 13-year period, the resorption of the adjacent bone, and healing following nonsurgical periodontal therapy. SRP is regarded as the cornerstone of periodontal therapy. Its effectiveness in the treatment of chronic periodontitis, when accompanied by good oral hygiene, has been repeatedly shown [23, 24]. Subgingival plaque and calculus can be substantially removed by SRP [17, 25, 26], creating a microenvironment that is favorable for tissue healing. In this case, initial periodontal therapy reduced pocket depths from a range of 5 to 10 mm to 4 mm. Randomized clinical trials indicate that SRP of molars leads to a 0.67 to 1.2 mm mean reduction of pocket depth at sites initially 4 to 6 mm deep and 0.94 to 2 mm reduction at sites initially deeper than 6 mm [24]. Isidor and Karring [27] reported a 3.7 mm reduction of pocket depth at sites with angular defects 12 months after SRP. The improvement observed in this case was consistent with these clinical studies.

The changes in alveolar bone density documented by this case were noteworthy. Although the serial radiographs were not standardized, they demonstrate that bone resorption occurred distal to tooth 47 during the period when calculus was present. An increase in bone density was evident two years after completion of periodontal therapy. While nonsurgical periodontal treatment typically leads to the formation of a long junctional epithelium [28], partial bone fill in an infrabony periodontal defect can occasionally occur following careful SRP. Hwang et al. [29] reported an increase in bone density in sites with more than 3 mm vertical bone loss after SRP. In a retrospective study focused on treatment of periodontal infrabony defects, SRP resulted in a 2.3 mm mean reduction of pocket depth, a 1.5 mm clinical attachment level gain, and a 0.7 mm reduction of infrabony defect depth with complete bone fill in some cases. Initial defect depth and use of adjunctive antibiotics were positively associated with a reduction of radiographic defect depth [30].

Previous studies [17, 18, 31] indicate that it is very difficult to attain complete removal of plaque and calculus in deep pockets. The amount of residual calculus is significantly correlated with pocket depth [17, 32]. Various methods, including flap exposure and pocket distention to gain access to diseased sites, can facilitate removal of subgingival calculus [33, 34]. The favorable treatment outcome observed in this patient could be attributable to several factors. The patient maintained a high level of oral hygiene throughout her treatment in our clinic. The level of oral hygiene achieved during the healing and maintenance phases following active treatment has a great impact on treatment outcome [35]. Adjunctive administration of azithromycin may have also enhanced the outcome. Although there is currently no standard protocol for antimicrobial chemotherapy in the treatment of periodontitis, the clinical benefits obtained from adjunctive systemic antimicrobials can justify their use in patients with deep pockets and progressive attachment loss [36]. Several controlled clinical trials have demonstrated that adjunctive use of systemic azithromycin can significantly enhance the clinical response to SRP, especially at sites with deep pockets. At sites with initial depths of ≥6 mm, adjunctive azithromycin with SRP resulted in pocket reduction that was approximately 0.7 to 0.9 mm greater than that produced by SRP alone [37, 38]. In smokers with chronic periodontitis sites initially deeper than 6 mm, the magnitude of pocket reduction gained by treatment with azithromycin and SRP was 1.54 mm greater than that obtained from SRP alone [39]. Azithromycin has a broad antimicrobial spectrum against a variety of periodontal pathogens [40]. It typically yields high therapeutic concentrations in gingival tissues and gingival crevicular fluid that are sustained for at least two weeks after the initial dose [41–43]. Its long half-life time allows a once-daily regimen, leading to better patient compliance. Azithromycin is concentrated inside host cells including fibroblasts and neutrophils, which may enhance the clearance of pathogens from diseased sites [44, 45]. Its anti-inflammatory activity, which is effective in the treatment of chronic inflammatory pulmonary diseases, could promote periodontal healing from severe inflammation [46–48]. Consistent with the patient's preference to avoid surgical treatment and potential undesirable consequences associated with ONJ, the rationale for utilizing azithromycin was to enhance the response to SRP. In this specific case, the potential benefits of azithromycin included suppression of periodontal pathogens in deep pockets, induction of anti-inflammatory activity, and promotion of healing through persistence at low levels in macrophages and fibroblasts in periodontal tissues [48]. The observed treatment outcome is consistent with a previous case series [49], in which bone regeneration and resolution of gingival inflammation were observed after a single course of azithromycin in combination with debridement.

While bisphosphonates are effective in conserving bone mass and bone trabecular thickness in patients with osteoporosis or osteopenia, their long-term side effects are sources of concern. In this case, a history of treatment with alendronate and concern about the potential risk of ONJ following extraction of tooth 47 had motivated the patient to investigate alternative treatment options. Alendronate has an estimated terminal skeletal half-life of 10.9 years, so its benefits and any side effects are prolonged [50]. While the risk of developing ONJ in patients who have taken relatively low oral doses of antiresorptive agents appears to be low, treatment plans that minimize periosteal and intrabony exposure or disruption are preferred [22].

It is clear that detection of oral disease at sites that are difficult to access can be challenging. Using tactile exploration alone, it is difficult to detect calculus on the distal aspect of a distally inclined second molar. Similarly, radiographic calculus detection can be undermined by the predominance of radiopacities associated with the bony architecture of the mandibular retromolar area. On the other hand, this patient had maintained good oral hygiene and visited her general dentist for recall appointments every six months for an extended period of time. It should have been possible to detect the presence of calculus, inflammation, deep probing depths, and bone loss at an earlier point in time. When an isolated site exhibits persistent signs of severe inflammation, all available diagnostic information should be analyzed to explain the underlying cause. In this case, radiographic diagnosis was ultimately facilitated by the availability of serial radiographs that clearly documented increased deposition of calculus over time. Based on these findings, it was possible to devise a conservative periodontal treatment plan that allowed the patient to retain all her teeth.
